# *Staphylococcus aureus* Lipase 3 (SAL3) is a surface-associated lipase that hydrolyzes short chain fatty acids

**DOI:** 10.1371/journal.pone.0258106

**Published:** 2021-10-07

**Authors:** Naren Gajenthra Kumar, Daniel Contaifer, Dayanjan S. Wijesinghe, Kimberly K. Jefferson

**Affiliations:** 1 Department of Microbiology and Immunology, School of Medicine, Virginia Commonwealth University, Richmond, Virginia, United States of America; 2 Department of Pharmacotherapy and Outcomes Sciences, School of Pharmacy, Virginia Commonwealth University, Richmond, Virginia, United States of America; Karl-Franzens-Universitat Graz, AUSTRIA

## Abstract

Bacterial lipases play important roles during infection. The *Staphylococcus aureus* genome contains several genes that encode well-characterized lipases and several genes predicted to encode lipases or esterases for which the function has not yet been established. In this study, we sought to define the function of an uncharacterized *S*. *aureus* protein, and we propose the annotation *S*. *aureus* lipase 3 (SAL3) (SAUSA300_0641). We confirmed that SAL3 is a lipase and that it is surface associated and secreted through an unknown mechanism. We determined that SAL3 specifically hydrolyzes short chain (4-carbon and fewer) fatty acids and specifically binds negatively charged lipids including phosphatidic acid, phosphatidylinositol phosphate, and phosphatidylglycerol, which is the most abundant lipid in the staphylococcal cell membrane. Mutating the catalytic triad S^66^-A, D^167^-A, S^168^-A, and H^301^-A in the recombinant protein abolished lipase activity without altering binding to host lipid substrates. Taken together we report the discovery of a novel lipase from *S*. *aureus* specific to short chain fatty acids with yet to be determined roles in host pathogen interactions.

## Introduction

Bacterial lipid metabolism can play diverse roles in host pathogen interaction either through its effects on the bacteria, (e.g. by contributing to bacterial fitness), or through its effects on the host (e.g. through modification of host lipids). Bacterial lipid modifying enzymes may be catabolic, anabolic, or they may transform the configuration of lipids without changing their total carbon content. Since *S*. *aureus* produces all three classes of enzymes, understanding the role of *S*. *aureus* lipid metabolism in modulating host-pathogen interactions is an area of active research. Catabolic enzymes, including lipase and esterase, hydrolyze complex lipids to release free fatty acids or small molecules that alter the host response to bacterial infection by degrading structural components such as phosphatidylinositol (PI) and associated GPI anchored proteins from the cell surface. Utilization of host derived fatty acids by bacterial anabolic enzymes can alter virulence factor expression as they esterify free fatty acids to available glycerol, cholesterol, sphingolipids and phospholipids and alter the fluidity of bacterial and/or host membranes [1–4]. Growing evidence suggests that activity of *S*. *aureus* lipases and esterases alter the innate immune response [5] and can alter the kinetics of viral replication in infected host cells [6] through unknown mechanisms. Characterization of the fatty acid chain length and associated lipid backbone of substrates targeted by these enzymes will be vital towards understanding the underlying mechanism of action.

*Staphylococcus aureus* is equipped with a number of lipid modifying enzymes, of which the most abundant secreted lipase is the triacylglycerol hydrolase, SAL2, also referred to as Geh (encoded by *geh*/*gehB*) [6–8]. Other lipases produced by *S*. *aureus*, including SAL1 (encoded by *gehA*) [8], PI-PLC [9], and the Fph family of enzymes [10], are unique in their function and substrate chain length specificity. The Geh family of enzymes, including SAL1 and SAL2, are specific for short (C2-C6) and medium-to-long (C8-C16 and greater) chain (respectively) fatty acid-associated neutral lipids like triglycerides [11]. Phospholipase C, which specifically hydrolyzes PI, was found to be important for survival of *S*. *aureus* in whole blood [9]. The two-component fatty acid kinase (composed of subunits FakA and FakB) mediates incorporation of host derived fatty acids by *S*. *aureus* [3,12,13]. Through this pathway, *S*. *aureus* can scavenge host lipids to alter bacterial lipid composition and virulence factor expression [4,7]. While recent work by others in the field has established the importance of lipases, more work on characterizing the specificity of these enzymes will help unravel the mechanism by which *S*. *aureus* lipases modulate the immune response. In this study, we investigated the activity and substrate specificity of an uncharacterized lipase using both classical and mass spectrometry-based approaches. To follow the nomenclature of previously characterized *S*. *aureus* lipases, SAL1 and SAL2, we propose the designation *S*. *a**ureus* lipase 3, or SAL3. The genes that encode SAL1 and SAL2 are *gehA* and *gehB*, respectively. In order to avoid confusion with *S*. *epidermidis* lipase genes *gehC* and *gehD*, we propose the designation *gehE* for the gene encoding SAL3.

## Materials and methods

### Recombinant His-tag protein expression and purification

*Escherichia coli* codon-optimized genes encoding SAL3 (old locus tag: SAUSA300_0641, new locus tag: SAUSA300_RS03435) and a quadruple mutant version (S^66^-A, D^167^-A, S^168^-A, and H^301^-A) were synthesized and cloned into the BamHI and XhoI sites of pUC57 by GeneWiz. The wildtype gene (5’-GGGGGATCCGATCAGATCACCAACGAAAATC-3’ and 5’-CCCCTCGAGTTAGTTCAGAGGATTCAGCGG-3’) and the quadruple mutant (5’-GGGGGATCC GATCAAATCACCAATGAAAAT-3’ and 5’-CCCCTCGAGTTAATTCAGCGGGTTCAGAG-3’) were amplified from plasmid DNA by PCR using primers that removed the signal peptide (predicted cleavage site Y/D 30–31 aa). The PCR products were digested with BamHI and XhoI and the DNA fragments were ligated to pET32a. CH3-Blue were transformed with the ligation reactions and plated on Luria Bertani (LB) agar plates containing 100 μg ampicillin/mL. Candidate clones were verified by sequencing. Plasmids with the correct sequence were transformed into BL21-CodonPlus (DE3)-RIPL cells (Agilent) and the recombinant wildtype SAL3 peptide (rSAL3) and the quadruple mutant (rSAL3MUT) were expressed by inoculating 1 L cultures of LB containing 100 μg ampicillin/mL and 35 μg chloramphenicol/mL with 60 mL of overnight cultures and incubating at 37°C in baffled Erlenmeyer flasks with shaking. These were grown to an OD_600nm_ = 0.5 and induced with 1 mM IPTG for 2 hr. Cells were collected by centrifugation, washed with 20 mL of 1X PBS, and frozen at -80°C. Frozen pellets were resuspended in 20 mL of Bacterial Protein Extraction Reagent (B-PER) containing a cOmplete EDTA-free mini protease inhibitor tablet (Sigma) and the bacteria were lysed by passing them through a French pressure cell three times at 750 psi. Unlysed bacteria and debris were removed by centrifugation at 3,000 rpm at 4°C for 30 min. Recombinant protein was purified by metal ion affinity using 1 mL HisPur cobalt resin (Fisher) as recommended by the manufacturer. Purified recombinant SAL3 was eluted with 3 mL 100 mM imidazole. Purified proteins were dialyzed against 1X PBS using 10,000 kDa molecular weight cutoff Slide-a-lyzer cassettes (Thermo).

### Immunofluorescence analysis of extracellular SAL3

Purified rSAL3 was sent to New England Peptides for production of polyclonal rabbit antiserum using their standard protocol.

*S*. *aureus* strain 6716, which is deficient for protein A production, was cultured in 20 mL or 100 mL of media in 250 mL baffled flasks overnight from a single colony. Bacteria were collected from 250 μL of the culture by centrifugation. The bacteria were fixed with 250 μL of 4% paraformaldehyde for 45 min at 21°C with end over end rotation, collected by centrifugation, and resuspended in 125 μL of 2% gelatin for 30 min at 21°C. Primary antisera (α-SAL3 or α-IsaB as a positive control for surface expression) was added at a final dilution of 1:25 and incubated 30 min at 21°C. The bacteria were collected by centrifugation at 10,000 rpm, washed 3 times with 100 μL of sterile 1X PBS and probed with Alexa Fluor 594-conjugated goat anti-rabbit antiserum. The bacteria were washed again and 10 μL of a 1:10 dilution of the cell pellet was dried onto a glass slide. A drop of DAPI-Prolong Gold was added, and the bacteria were imaged using a 40 X objective on an Olympus fluorescence microscope.

### Subcellular localization of SAL3 by Western analysis

The *gehE* gene was disrupted in *S*. *aureus* strain 6716 by transpositional insertion through phage 80α-mediated transduction of the mariner transposon from Nebraska Transposon Mutant Library clone NE104. *S*. *aureus* 6716*gehE*::Tn was cultured for 4 hours at 37°C in 3 mL of TSB in 50 mL conical tubes with shaking at 250 rpm. Bacteria were collected by centrifugation and the spent medium was filtered through a 0.45 micron syringe filter and reserved. The bacteria were washed three times with 1 mL of sterile 1X PBS and resuspended in 250 μL PBS. The spent medium and bacterial cell fractions were analyzed by denaturing polyacrylamide gel electrophoresis (PAGE) using the NuPAGE Bis-Tris system (Thermo) wherein the bacteria were lysed directly in NuPAGE LDS sample buffer (2X final concentration) and sample reducing agent (1X final concentration) by heating to 80°C for 30 minutes followed by sonication. Following PAGE, the proteins were transferred to 0.2 *μ*m PVDF. The PVDF membranes were blocked in 5% BSA in PBS overnight at 4°C. The blots were probed with SAL3 antiserum at a 1:10,000 dilution in 1X PBS containing 0.05% Tween 20 (1X PBST) and 0.5% BSA at 21°C for 1 hr. The membranes were washed three times in 1X PBST and probed with a 1:10,000 dilution of goat anti-rabbit HRP tagged secondary antibody. The membranes were washed as above, developed using the ECL Prime system (Cytiva), and analyzed using a ChemiDoc system (Bio-Rad).

### Bacterial surface shaving method

To determine whether or not SAL3 is expressed on the outer surface of *S*. *aureus*, we used a surface protein shaving method [14]. Bacteria were collected and resuspended in PBS containing 2 mg proteinase K/mL and incubated for 30 minutes at 37°C. The bacteria were collected by centrifugation, washed in PBS, and analyzed by Western as described in the previous section.

### Quantitative lipase activity assays

Lipase activity of rSAL3 and rSAL3MUT was assayed using the QuantiChrom^™^ DTNB based lipase assay kit (BioAssay Systems) [15] as per manufacturer instructions. To test the effect of calcium on SAL3, lipase activity was measured using rSAL3 that had been treated with 10 mM EDTA prior to dialysis against 1X PBS to strip any bound Ca^2+^ ions from the protein. The EDTA treated rSAL3 was either tested alone or supplemented with 1 mM CaCl_2_.

Paranitrophenol assays were performed using esters of paranitrophenol with varying length of fatty acids. Paranitrophenol esters butyrate and octanoate were solubilized in 100% acetonitrile to a concentration of 100 mM and diluted to the final concentration of 2 mM in assay buffer (100 mM Tris HCl 100 mM NaCl at pH 7). Long chain fatty acid containing esters were solubilized in 4:1 IPA:ACN to a final concentration of 100 mM. Specific activity of the enzyme was calculated from the amount of ester hydrolysis that occurred in the presence of 0 μg, 2 μg, 4 μg, 6 μg, 8 μg rSAL3 or rSAL3MUT in a final reaction volume of 200 *μ*l during a 20 minute incubation at 37°C. Ester hydrolysis was measured at 405 nm, and release of paranitrophenol was determined based on a standard curve prepared for paranitrophenol.

### Analysis of *1-oleoyl 2-acetyl* glycerol by LC-MS

To prepare 2 μg 1-oleoyl 2-acetyl glycerol/mL (OAG), a 2 mg/mL stock solution in chloroform was diluted 1:100 in 100% methanol, and this was diluted 1:10 in 1x PBS. To assess hydrolysis, 0.2 mM of rSAL3 in 44 μL of 1X PBS was incubated with 5 μL of the 2 μg/mL stock for 2 hr at 37°C with gentle agitation. Hydrolysis of OAG was determined by LC-MS/MS analysis. At the end of 2 hours 200 μL of methanol was added to the mixture to stop the reaction followed by the addition of 20 μL of SPLASH LIPIDOMIX internal standard mix (Avanti) containing a DAG internal standard (DG 15:0–18:1(d7)) (10 μg/mL) and 20 μL of deuterated oleic acid internal standard (α-oleic acid d17). Analysis of OAG and free oleic acid release was determined by reverse phase chromatography using a Waters CSH C18 (2.1x100 mm) column. Mobile phase A 60:40 Acetonitrile:Water 10 mM Ammonium acetate 0.1% Formic acid, Mobile phase B 90:10 Isopropanol: Acetonitrile 10 mM Ammonium acetate 0.1% Formic acid. Elution of analytes was performed using a flow rate of 0.6 mL/min over the following gradient 0min- 15%, 2min- 30%, 2.5min-48%, 11min-82%, 11.5min- 99%, 13.5min- 99%, 13.6min-15%, 15.6min- 15%. Mass spectrometry was performed on an ABSCIEX QTRAP 6500+ TEM 500°C, CAD -1, CUR 20, GS1 60, GS2 40, IS 4500, CXP 10, EP 10, DP 60, CE for OAG (20) and IS DAG CE 30 in the positive mode for the analysis of diacylglycerol. Analysis of free fatty acid release was performed simultaneously in the negative mode, FFA 18:1 and IS- FFA 18:1-d17 was determined based on accurate mass. CE -10.

### Analysis of free fatty acids release by LC-MS

Bovine heart extract micelles were prepared as previously described [16] and 250 μL of the micelles were incubated with 10 μg/mL of rSAL3 or rSAL3MUT for 20 hours at 37°C. Total lipid extraction was performed by Bligh/Dyer [17] method. Briefly, 250 μL of the suspension was added to 1 mL of methanol and 0.5 mL of chloroform and vortexed for 5 seconds. This mixture was combined with 1 mL of chloroform and vortexed before adding 1 mL of water to induce phase break. The tubes were centrifuged at 3,200 rpm for 10 minutes at 4°C. The lower organic phase was transferred to a fresh glass test tube and dried under vacuum for 1 hour. The dried lipids were resuspended in 100 μL of ethanol and stored at -20°C until analysis by LC-MS. Free fatty acid analysis was performed on a Waters BEH HILIC 2.1x100 mm column over 12 minutes. Gradient separation was performed using mobile phase A: Water (15 mM Ammonium acetate) and Mobile phase B: Acetonitrile over 12 minutes. 1 minute- 4% B, 3 minute- 60% B, 5 minute- 70% B, 8 minute- 80% B, 10–12 minute-4% B.

### Protein lipid overlay assay

Protein lipid overlay assays to determine the interaction of SAL3 with cell membrane-associated lipids was performed using Echelon lipid strips. Lipid strips were blocked overnight at 4°C in blocking buffer (10mM Tris Base, 150 mM NaCl pH 8 1% Tween 20, 3% Bovine serum albumin (BSA)). The strips were washed in 10 mL wash buffer (10 mM Tris Base, 150mM NaCl pH 8, 1% Tween 20) 3X for 5 min each with shaking at 200 rpm at 21°C and probed with 10 mL blocking buffer containing purified, recombinant SAL3 at a concentration of 10 μg/mL at 21°C for 1 hr with shaking. The strips were washed with 10 mL of the wash solution 3X. The strips were incubated in blocking buffer containing SAL3 rabbit antiserum at a dilution of 1: 5,000 at 21°C for 1 hr and washed. To detect the bound antibody, the strips were incubated in horseradish peroxidase-labelled goat anti-rabbit secondary antibody at a final dilution of 1: 100,000 in blocking buffer at 21°C for 1 hr, washed, and developed using ECL Prime reagents. Imaging was performed using a Biorad gel dock with auto exposure settings. Strips with OAG were made in-house as follows. Stocks of 2 μg/mL OAG were prepared in a solution containing 25% chloroform, 50% methanol, 25%water, 1% Ponceau stain and 2 μL of the prepared solution was dried onto PVDF. The strips were analyzed for SAL3 binding as above.

## Results

### Similarity of SAL3 to other serine hydrolases

**Fig 1:**
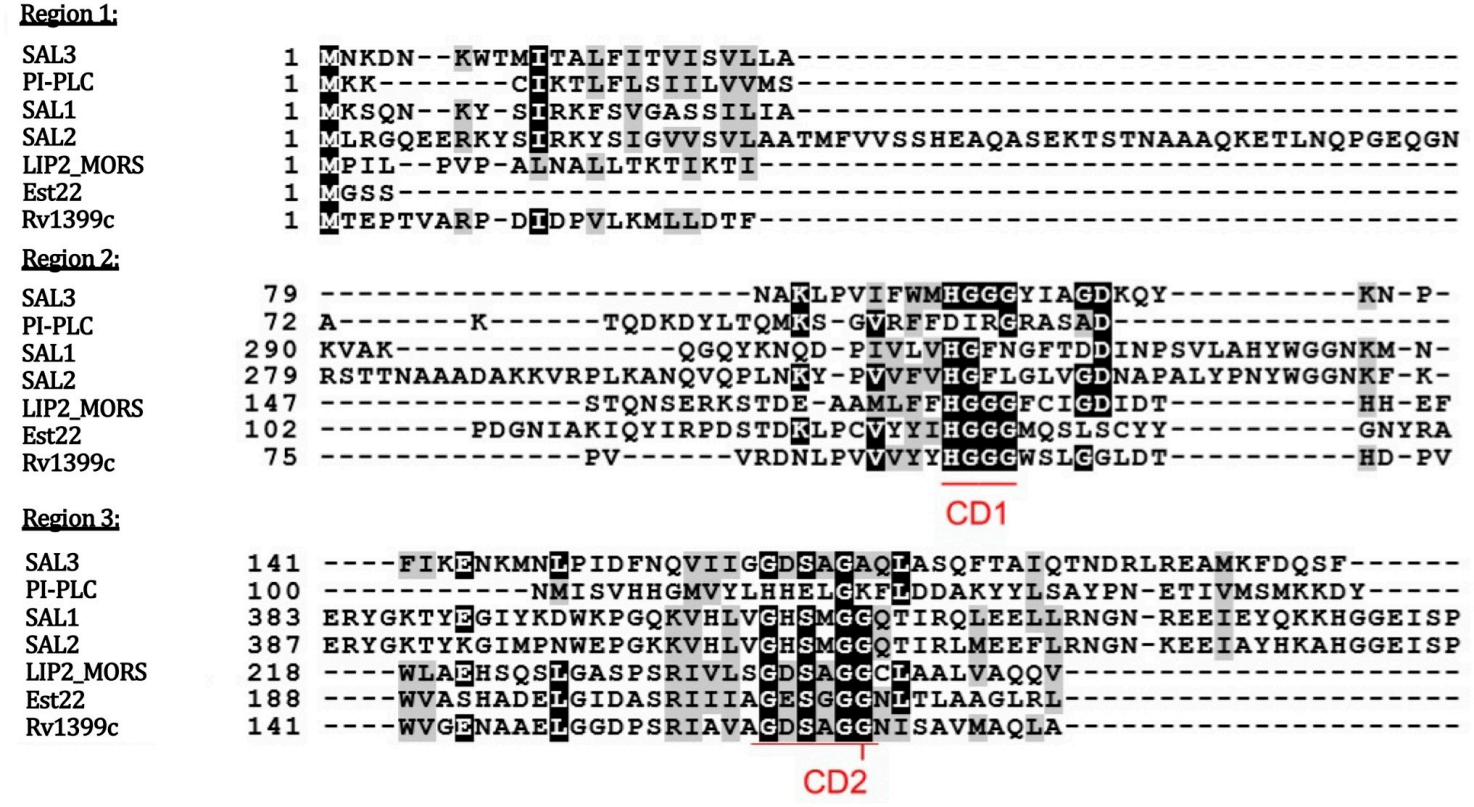
Multiple sequence alignment of SAL3. Multiple sequence alignment of SAL3 (SAUSA300_0641) with other characterized lipases of *S*. *aureus* (PI-PLC, SAL1(SAUSA300_2603), SAL2(SAUSA300_0320)) and bacterial esterase Lip2_MORS1 (*Moraxella*), Est22 (Deep sea isolate), Rv1399c (*Mycobacterium tuberculosis*).

Multiple sequence alignment of SAL3 with other known staphylococcal and bacterial lipases was performed to identify regions likely to contribute to the catalytic activity of these enzymes. SAL3 was aligned to characterized enzymes from *S*. *aureus* (SAL1, SAL2 and PI-PLC) and other bacterial species, including Moraxella (Lip2_MORS1), Rv1399c [18] (*Mycobacterium tuberculosis*) and Est22 [19] (deep sea isolate) to map regions important for catalytic activity. For some enzymes like Est22, a crystal structure is available and enabled the identification of putative active positions in the predicted model of the protein. As expected, the greatest similarity was found between *S*. *aureus* lipases SAL1 and SAL2 (45.05%). SAL3 shares 20–22% similarity with enzymes Est22, Lip2_MORS1 and Rv1399c. Similarity between candidates was restricted to 3 distinct regions characteristic of the class of enzymes containing the alpha/beta hydrolase fold domains. Since most staphylococcal lipases are expressed with signal sequences that are proteolytically cleaved post-synthesis for the activation of lipase activity, similarities were observed in the first 23 amino acid residues of only *S*. *aureus* proteins (Region 1 Fig 1). In addition to the signal sequence, similarities were observed in the core region of the enzymes near the catalytic serine residue essential to the activity of lipases (Region 3 Fig 1). Significant similarity was observed between all proteins in Region 2 with the presence of the HGGG motif found to be characteristic of microbial lipases [20,21].

### SAL3 is an extracellular surface-associated protein

**Fig 2:**
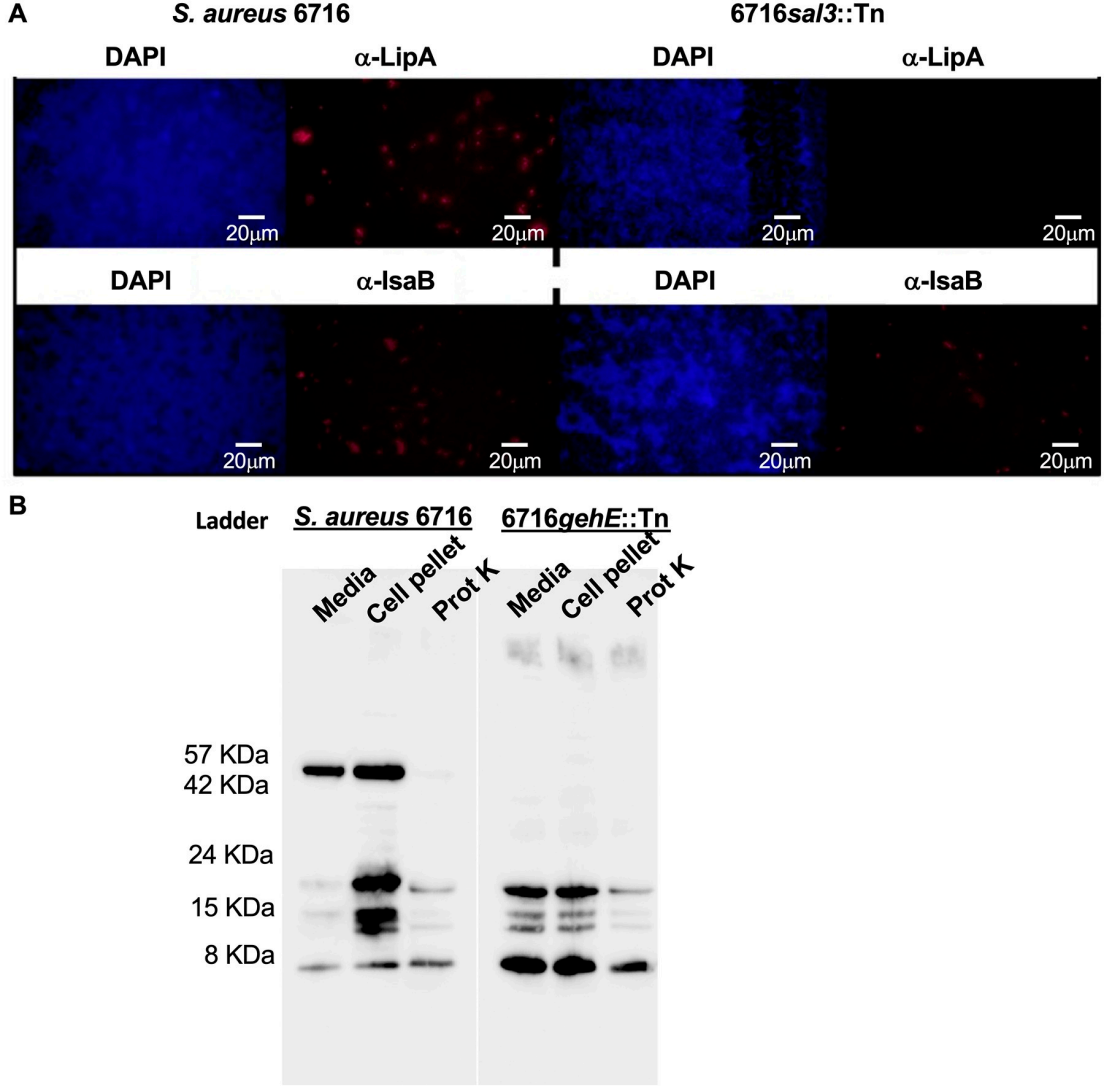
SAL3 is extracellular and associated with the cell envelope. **A.** Localization of SAL3 in wild type *S*. *aureus* strain 6716 (protein A deficient) and mutant 6716*gehE*::Tn was determined using primary polyclonal rabbit antisera raised against recombinant protein and detection by immunofluorescence using an Alexa Fluor 594-conjugated secondary antibody. IsaB antisera was used as a positive control for a surface-associated protein. DAPI was used to stain DNA inside the bacteria. **B.** Western blot of SAL3. From left to right: Media: Spent medium from culture of protein A-deficient S. aureus strain 6167. Cell pellet: 6167 cells lysed directly in denaturing PAGE buffer. Prot K: 6167 cells treated with proteinase K (shaved). Media: Spent medium from 6716*gehE*::Tn culture Cell pellet: 6716*gehE*::Tn cells Prot K: 6716*gehE*::Tn treated with proteinase K.

To determine the subcellular localization of SAL3, an immunofluorescence assay was performed. We used the protein A-deficient strain, *S*. *aureus* 6716 to avoid non-specific interaction between the antibody probes and protein A on the bacterial surface. Bacteria from overnight cultures of *S*. *aureus* 6716 and 6716*gehE*::*Tn* were collected, and non-permeabilized bacteria were probed with rabbit antiserum specific for SAL3 and an Alexa Fluor 594-labeled secondary antibody. The immunodominant staphylococcal antigen B, which is surface-associated [22], was probed on 6716 and 6716*sal3*::*Tn* as a control. SAL3 was detected on non-permeabilized WT 6716 but not 6716*gehE*::*Tn* suggesting that the immunofluorescence method was specific for SAL3, and that SAL3 was present on the surface of the WT bacteria. No difference in the expression of IsaB was observed in WT and 6716Δ*gehE*::*Tn*, suggesting that inactivation of *gehE* by transpositional insertion did not universally affect the expression of other surface associated proteins (Fig 2A). Western blot analysis was used to confirm secretion of SAL3 and expression on the external surface of bacteria. The protein was detected in spent media from cultures and on whole cells. Shaving of the bacterial cell surface with proteinase K resulted in the loss of SAL3, further supporting the conclusion that SAL3 is secreted and that at least a portion of it is localized to the external cell surface (Fig 2B).

### SAL3 is a serine hydrolase

**Fig 3:**
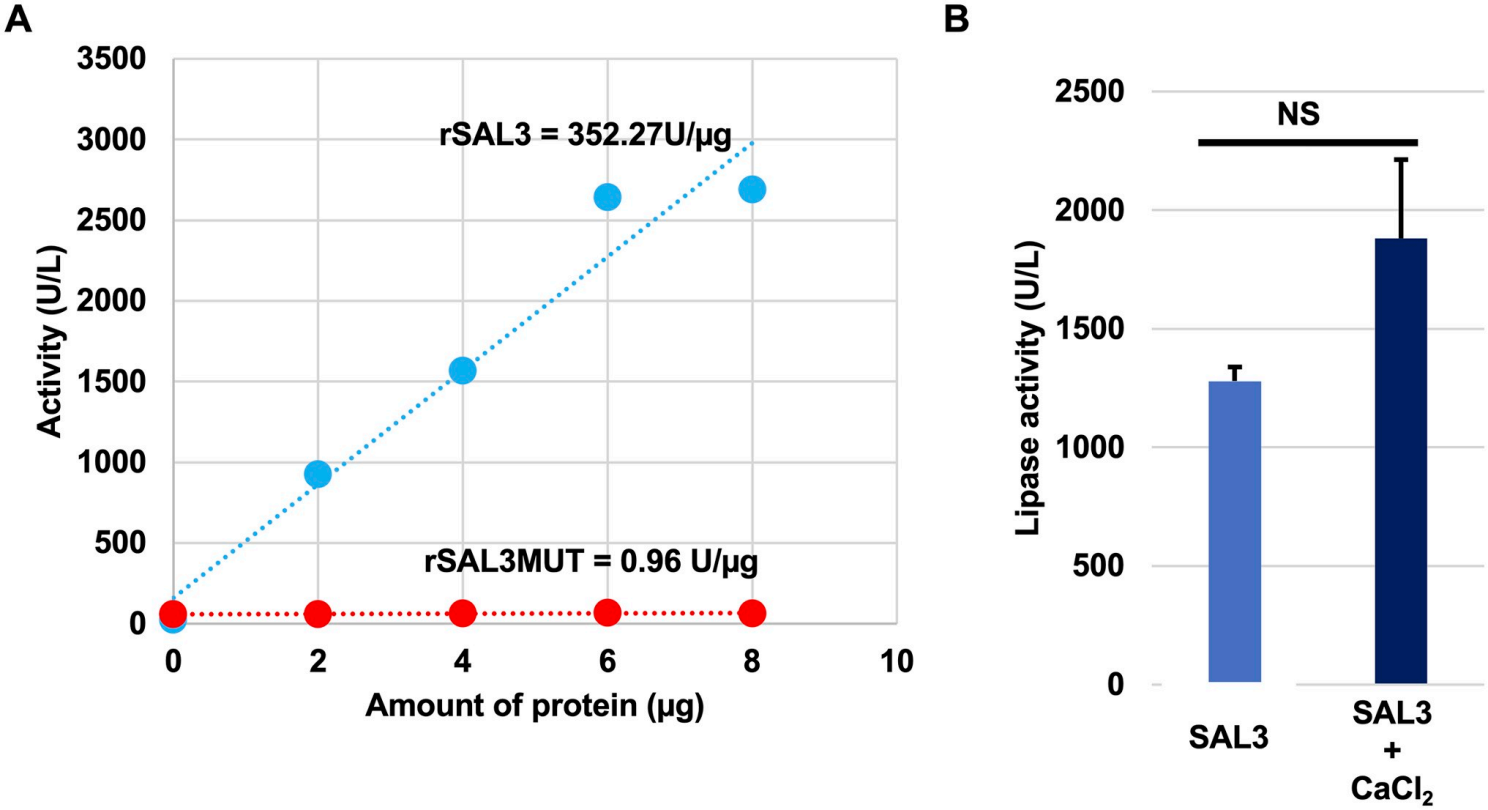
Lipase activity of SAL3 depends upon conserved residues but does not require calcium. **A.** The lipase activity of recombinant SAL3 (rSAL3) and a version of the peptide in which Ser^66^, Asp^161^, Ser^162^, and His^295^ had all been converted to alanine residues (rSAL3MUT), was determined using a DTNB based lipase assay kit. **B.** The effect of calcium on activity of 10 μg rSAL3/mL (0.25μM) was determined by removing calcium by dialysis against PBS containing EDTA and then adding 1 mM calcium back to the protein. Error bars are standard deviations of experiments done in triplicates. Significance for t-test p< 0.05.

SAL3 was expressed recombinantly in *E*. *coli* and purified to yield rSAL3. A mutant form of SAL3, in which Ser^66^, Asp^161^, Ser^162^, and His^295^, all predicted to be essential for lipase activity based on studies of other lipases, were converted to alanine residues was designated rSAL3MUT. Using a DTNB based assay, we found that rSAL3 had lipase activity but the activity was absent or severely abrogated in rSAL3MUT (Fig 3A). As catalytic activity of lipases is often enhanced by the binding of divalent cations to the C-terminal calcium binding site, we assessed the influence of calcium by first chelating cations associated with rSAL3 with EDTA and the adding Ca^2+^ back (Fig 3B). No significant increase in lipase activity was observed when CaCl_2_ was added to recombinant protein suggesting that the catalytic activity of SAL3 was not dependent on the presence of divalent cations and that SAL3 is distinct from other previously characterized *S*. *aureus* lipases with distinct C-terminal calcium binding sites [8,11].

### Substrate specificity of SAL3

**Fig 4:**
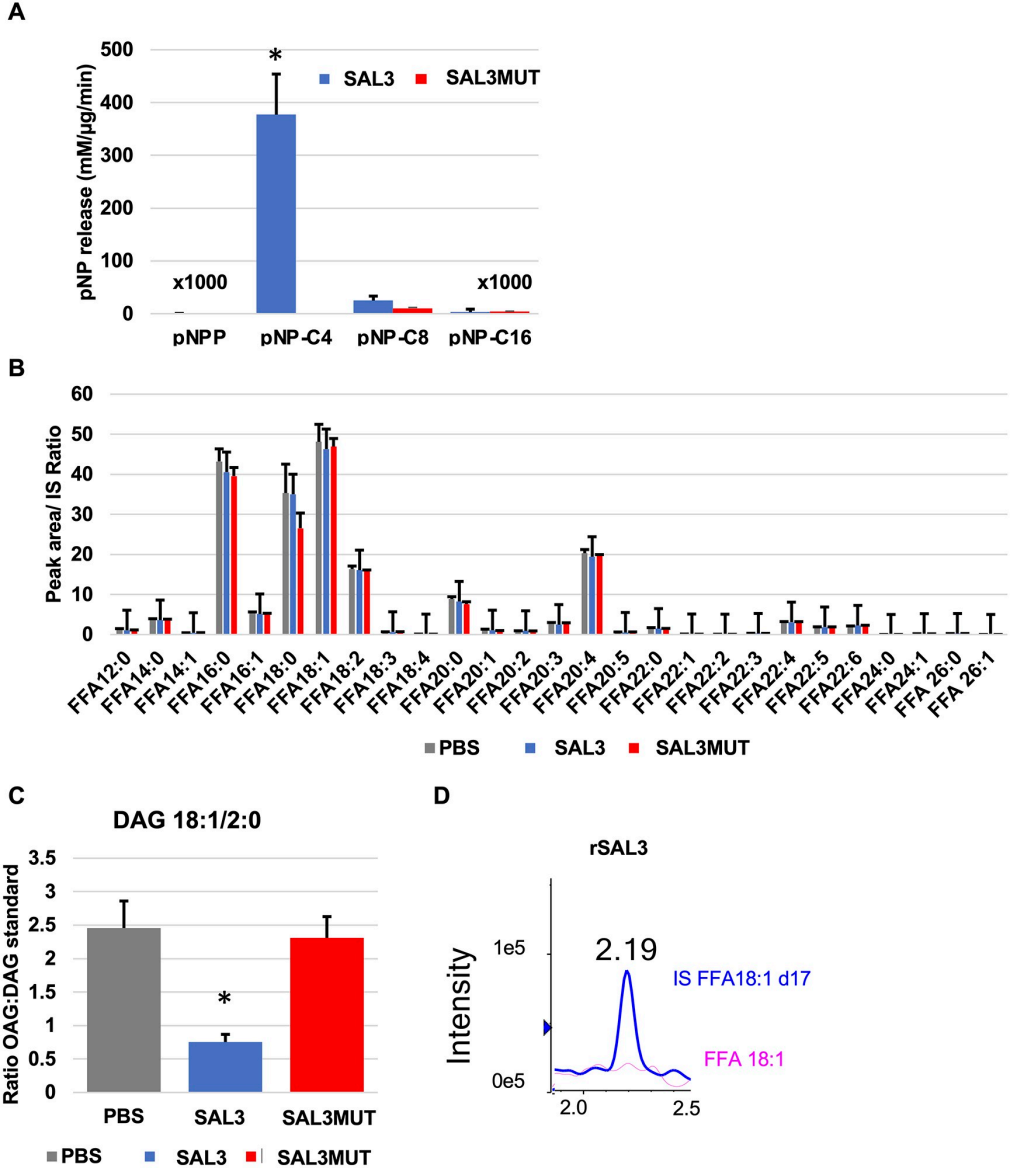
Fatty acid chain length specificity of SAL3. **A.** Specific activity of 10 μg rSAL3/mL (0.25 μM) and 10 μg rSAL3MUT/mL (0.25 μM) for hydrolysis of paranitrophenol esters with fatty acids of increasing chain length: pNPP (paranitrophenol phosphate), pNP-C4 (paranitrophenol butyrate), pNP-C8 (paranitrophenol octanoate), pNP-C16 (paranitrophenol palmitate). Error bars represent standard deviation of reactions carried out in triplicate. **B.** Analysis of free fatty acid release from bovine heart extract micelles by 10 μg rSAL3/mL (0.25 μM) or 10 μg rSAL3MUT/mL (0.25μM)/mL (0.25 μM). Recombinant proteins were in PBS and PBS alone was used as a control **C.** Hydrolysis of 2 μg 1-oleoyl 2-acetyl glycerol (OAG)/mL by 80 μg rSAL3/mL (2 μM). The y-axis shows the ratio between OAG and the internal standard DAG 15:0–18:1(d7). **D.** The red line shows that there was no detectable release of free oleic acid (FFA 18:1) in the reaction from panel C. An internal standard is shown in blue and was included as a reference for retention time. The x-axis is indicative of the retention time in minutes.

Synthetic substrates like fatty acid esters of paranitrophenol provide a method of rapid identification of fatty acid chain length preference of enzymes with unknown function. SAL3 exhibited greater catalytic activity against paranitrophenol esters with short chain fatty acids (C2-C4) and activity rapidly dropped off with increasing chain length (C8-C16) (Fig 4A). To test whether the activity was specific for the hydrolysis of short chain O-linked fatty acid esters, activity against paranitrophenol phosphate, a substrate for screening for phosphatase activity, was determined. The rSAL3 exhibited little or no activity against pNP-phosphate. Alanine substitution mutations introduced in rSAL3MUT abolished the activity against pNP-C4 (Fig 4A). The lack of hydrolysis of long chain fatty acids was also confirmed in an assay where micelles of bovine heart extract were treated with rSAL3 or rSAL3MUT and free fatty acids produced by enzymatic hydrolysis were monitored by mass spectrometry (Fig 4B). Treatment of the micelles with rSAL3 did not result in their degradation and release of long chain free fatty acids (C10 –C26:1). Thus, SAL3 is specific for the hydrolysis for short chain fatty acid containing soluble esters.

To further characterize the specificity of SAL3 for short chain fatty acid associated esters, the short chain fatty acid associated diacylglycerol, 18:1/2:0 (*1- oleoyl 2-acetyl glycerol* (OAG)) was used as a substrate. LC-MS was utilized and quantification of OAG hydrolysis and release of free fatty acid 18:1 were performed simultaneously. The rationale was that a reduction in the level of OAG would either be associated with the hydrolysis of the long chain fatty acid ester or the acetate from the sn-2 position. While the hydrolysis of the substrate in the presence of FFA 18:1 release would suggest specificity of the enzyme for long chain fatty acid hydrolysis, a reduction in OAG in the absence of long chain fatty acid release could only be due to the hydrolysis of the short chain acetate. When DAG 18:1/2:0 was incubated with rSAL3 and the rSAL3MUT, there was a marked reduction in the substrate only in reactions containing WT rSAL3 (Fig 4C). This was not accompanied with the release of FFA 18:1 (Fig 4D), suggesting that rSAL3-mediated degradation of DAG 18:1/2:0 (OAG) was due to the hydrolysis of the short chain acetate.

### Interaction of SAL3 with membrane lipids

**Fig 5:**
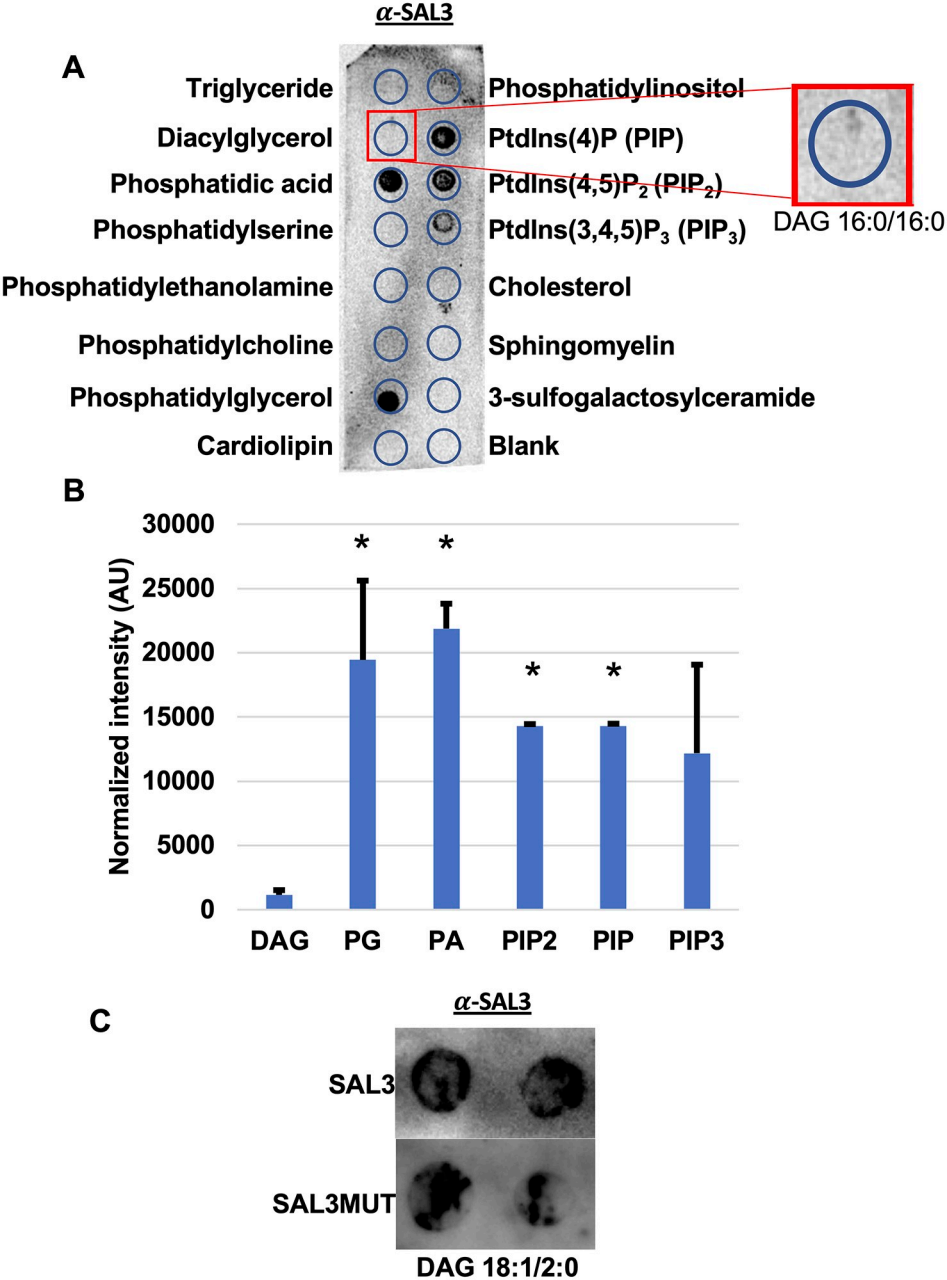
Interaction of SAL3 with membrane lipids. The interaction of SAL3 with membrane lipids was determined using protein lipid overlay assays. **A.** Lipids to which rSAL3 and rSAL3MUT bound were observed by chemiluminescence. **B.** Normalized intensity relative to background signal was assessed semi-quantitatively by ImageJ. * p< 0.05. **C.** A lipid overlay assay was designed to assess the interaction between rSAL3 and 1-oleoyl 2-acetyl glycerol (DAG 18:1/2:0). The two spots are technical replicates.

We reasoned that SAL3 would bind preferentially to its preferred substrate and to test this and determine whether it binds to other lipids found in mammalian cells, protein lipid overlay assays were performed. Purified rSAL3 interacted specifically with negative lipid classes like phosphatidylglycerol, phosphatidic acid and species of phosphatidylinositol (Fig 5A–5C). Limited binding of rSAL3 was observed with sphingomyelin (SM), and phosphoinositide 3, 4, 5 phosphate (PIP3). Furthermore, rSAL3 and rSAL3MUT bound to DAG 18:1/2:0 (Fig 5D) but not to DAG 16:0/16:0 on the Echelon lipid strips (Fig 5B) further supporting its specificity for short chain fatty acids.

## Discussion

Lipases play a major role in maintaining lipid homeostasis and the response to changing environments by altering lipid composition. Several lipases produced by *S*. *aureus* have been characterized but there are a number of genes predicted to encode lipases that have not yet been characterized. In this study, we initiated the characterization of a novel *S*. *aureus* lipase that we propose to name SAL3. SAL1 and SAL2 both contain a YSIRK motif within the signal sequence, which directs them for septal secretion [23,24]. In contrast, SAL3 lacks the YSIRK motif in the signal peptide region, but still contains a signal peptide for secretion. It also lacks an LPXTG motif for sortase-mediated cell wall anchoring, but its expression on the bacterial cell surface has been previously reported [25] and our data confirm expression during exponential phase and cell-surface association. Lipid binding assays demonstrated that SAL3 binds to phosphatidylglycerol, which is abundant in the *S*. *aureus* plasma membrane [26], and this could explain the retention of SAL3 on the surface of *S*. *aureus*.

As expected, based on amino acid similarity with other lipases, recombinant purified SAL3 exhibited activity in a chromogenic assay. Activity did not depend on the presence of calcium, and mutation of residues conserved in lipases (Ser^66^, Asp^161^, Ser^162^, and His^295^) to alanine residues resulted in complete loss of catalytic activity of SAL3. *S*. *aureus* SAL3 exhibits similarity with Rv1399c and Est22, which are short chain fatty acid-specific esterases, and similarly, rSAL3 was also specific for short chain fatty acids. Furthermore, SAL3 exhibited specificity towards *O-linked* fatty acid esters as it showed no activity against paranitrophenol phosphate (pNPP), a commonly used substrate for determining phosphatase activity.

Recombinant SAL3 was tested for its ability to hydrolyze the endogenous substrate, DAG 18:1/2:0 (OAG), previously shown to be important in initiating the cascade of phosphoinositide signaling important in mediating cell migration by upregulating leukotriene production independent of protein kinase C induction [27]. The binding of native and mutant recombinant SAL3 to OAG and its hydrolysis without the release of long chain free fatty acid confirmed the function of SAL3 as a short chain fatty acid-specific lipase and suggested that the enzyme could alter pools of this important lipid in vivo. Attempts to quantify the release of acetic acid (*sn2-*C2) from OAG hydrolysis by performing charge reversal derivatization using 2-picolylamine [28–30] were unsuccessful, likely due to the volatility of the short chain fatty acids. Instead, release of *sn2-*C2 was assessed indirectly by demonstrating the lack of release of the long chain fatty acid, 18:1. The data confirmed that rSAL3 hydrolyzed OAG but 18:1 was not released, suggesting that the *sn2-*C2 was released.

Diacylglycerols are abundant in *S*. *aureus* plasma membranes and are required to anchor bacterial cell envelope components [31]. An inability to properly anchor the bacterial cell envelope to the plasma membrane may increase sensitivity to environmental stimuli and cell wall disrupting agents. While the function of SAL3 for bacterial metabolism remains to be determined, its substrate specificity appears to partially overlap SAL2, in that it is catalyzes short chain fatty acid hydrolysis. Even though there is overlap in their preference for fatty acids, it is possible that SAL2 and SAL3 have different preferences for lipid backbones containing short chain fatty acids. It is also likely that there are other substrates in addition to OAG that are hydrolyzed by SAL3. Identification of the full spectrum of its activity will likely help to elucidate the biological function of SAL3 in more detail.

## Supporting information

S1 Fig Uncropped/Unaltered Western blot of SAL3 (from Fig 2). Lane 1: Gold-Bio BLUEstain molecular weight marker (not visible). Lane 2: Spent medium from culture of protein A-deficient S. aureus strain 6167. Lane 3: 6167 cells. Lane 4: 6167 cells treated with proteinase K (shaved). Lane 5 and 6: Empty wells. Lanes 7 and 8: 6716Δ*gehE*::Tn treated with proteinase K. Lane 9: 6716Δ*gehE*::Tn cells. Lane 10: Spent medium from 6716Δg*ehE*::Tn culture. Note that in Fig 2 in the manuscript, Lanes 8–10 have been reversed (mirror image) for clarity.
(TIFF)
