# Supplementary figures and images for: Staphylococcus aureus Lipase 3 (SAL3) is a surface-associated lipase that hydrolyzes short chain fatty acids
Source: PLoS One. 2021 Oct 7;16(10):e0258106. doi: 10.1371/journal.pone.0258106 (PMC8496776; doi:10.1371/journal.pone.0258106)

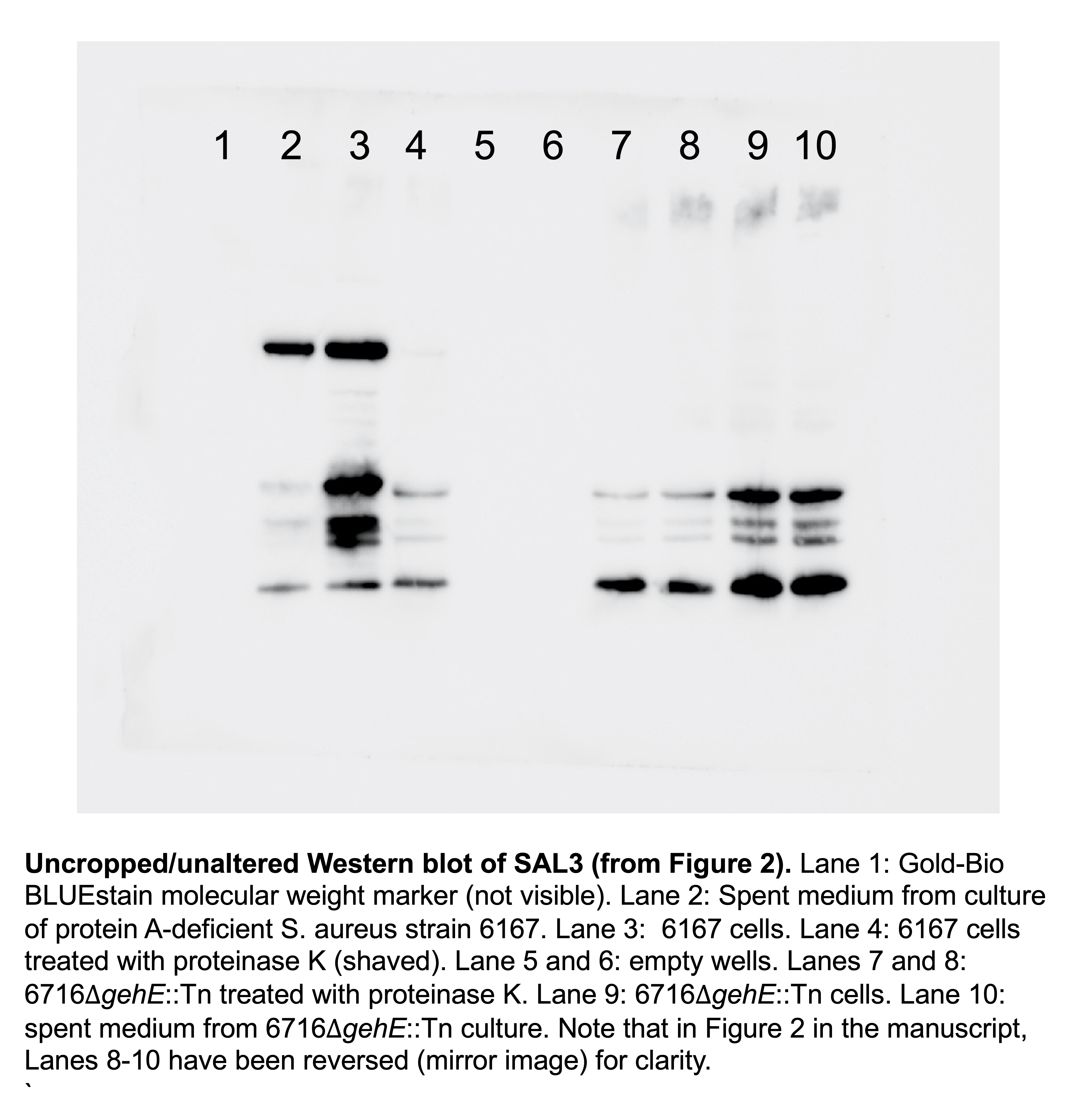

Supplement: S1 Fig — Lane 1: Gold-Bio BLUEstain molecular weight marker (not visible). Lane 2: Spent medium from culture of protein A-deficient S. aureus strain 6167. Lane 3: 6167 cells. Lane 4: 6167 cells treated with proteinase K (shaved). Lane 5 and 6: Empty wells. Lanes 7 and 8: 6716ΔgehE::Tn treated with proteinase K. Lane 9: 6716ΔgehE::Tn cells. Lane 10: Spent medium from 6716ΔgehE::Tn culture. Note that in Fig 2 in the manuscript, Lanes 8–10 have been reversed (mirror image) for clarity. (TIFF) [file pone.0258106.s001.tiff]
